# Fungal Aquaporins in Ectomycorrhizal Root Water Transport

**DOI:** 10.3389/fpls.2020.00302

**Published:** 2020-03-19

**Authors:** Hao Xu, Janusz J. Zwiazek

**Affiliations:** ^1^Summerland Research and Development Centre, Agriculture and Agri-Food Canada, Summerland, BC, Canada; ^2^Department of Renewable Resources, University of Alberta, Edmonton, AB, Canada

**Keywords:** hypha, major intrinsic protein, plant–fungal interaction, symplastic pathway, transport capacity

## Abstract

Ectomycorrhizal fungi influence root water transport of host plants. To delineate the exact mechanisms of how fungal partner alters root water relations, it is important to understand the functions of fungal transmembrane water channels, i.e., aquaporins, the key component in the symplastic pathways. In this paper, we discussed what roles the fungal aquaporins may play in root water transport. We also highlighted the opportunities of using integrated approaches to address rising questions in future hotspots of aquaporin and root water relations research.

## Impacts of Ectomycorrhiza on Root Water Relations

Ectomycorrhizal (EcM) fungi develop mutualistic associations with roots of Pinaceae and many hardwood species. Through their highly specialized structures, EcM fungi supply mineral nutrients and water to the roots of host plants in exchange for photosynthates.

**FIGURE 1:**
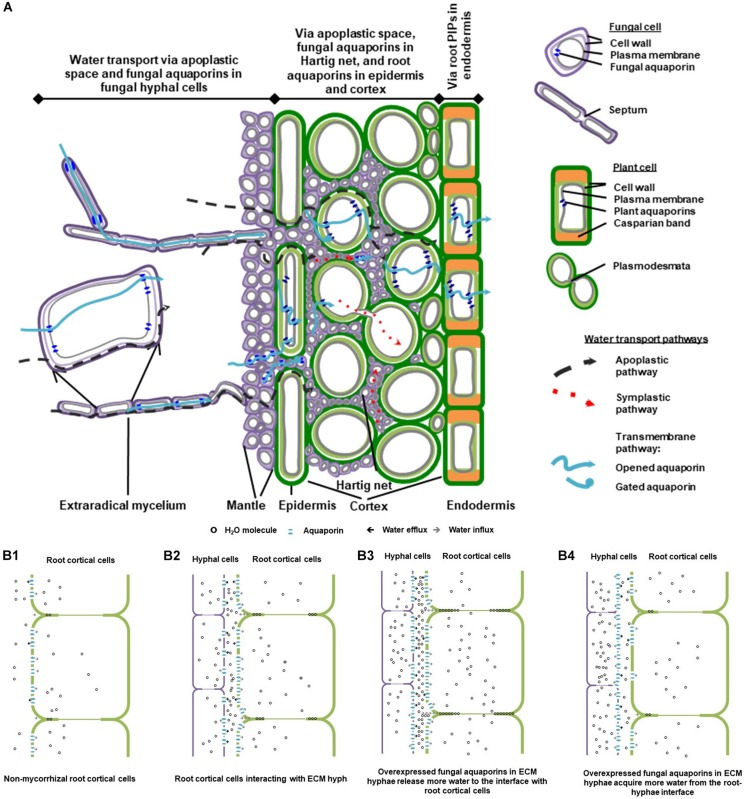
A schematic diagram of water transport pathways and aquaporin involvement in ectomycorrhizal (EcM) association. **(A)** A conceptual model of water pathways through EcM hypha-root continuity. The diagram was drawn based on the composite model of root water transport (Steudle and Peterson, 1998), with reference to that of the EcM root water transport suggested by Lehto and Zwiazek (2011). **(B)** Involvement of fungal and plant aquaporins in water transport at the hypha-root interface in EcM. The simplified model is postulated according to a study on the roles of fungal aquaporin *Laccaria bicolor* JQ585595 in EcM interaction and root hydraulic dynamics of the host plant *Picea glauca* (Xu et al., 2015). **(B1)** Water is transported in apoplastic space and cell-to-cell pathways in cortical cells in non-mycorrhizal root tips; **(B2)** In mycorrhizal root tips, root water transport in apoplastic, and cell-to-cell pathways is altered by the presence of mycorrhizal hyphae, as water released from mycorrhizal hyphae increases the hydration in the intercellular space of cortical cells, and the root aquaporins are engaged for transmembrane water transport; **(B3)** When transcript abundance of the fungal aquaporins is up-regulated, a moderate increase in fungal aquaporins contributes to the increase in water efflux from hyphal cells and in hydration of root intercellular space, which leads to further enhancement of apoplastic water availability to root; **(B4)** Conditionally, increased abundance of fungal aquaporins may cause more water influx into hyphal cells and less water in intercellular space available for root transport. The diagrams were modified from Xu (2015) with the author’s permission.

EcM fungi colonize the lateral roots extracellularly at the water permeable root tips, typically forming three compartments that alter water transport in the rhizosphere distinctly. They are the extraradical network of free-living mycelia, mantle sheath and Hartig net. The free-living fungal mycelia extend by cell elongation and infinite cell division, into an extensive extraradical network in the soil (Smith and Read, 2008). The hyphae at the growing mycelial front are actively involved in water and nutrient acquisition. Some hyphae go through different degrees of morphological differentiation and form specialized rope-like strands called rhizomorphs for medium-distance and long-distance soil exploration (Agerer, 2001). Vessel hyphae in rhizomorphs are enlarged with highly modified or absent septa that facilitate efficient water movement. Some peripheral hyphae in rhizomorphs display thickened and pigmented cell walls that may help to reduce water loss (Agerer, 2001; Peterson et al., 2004). Nutrients and water are transported along rhizomorphs to two structures at hyphae-root interface – the hyphal mantle sheath that envelops the root tip and forms an outwardly sealed compartment mainly for storage, and the intercellular Hartig net of hyphae surrounding the plant epidermal and outer cortical cells for substrate exchange (Figure 1A; Smith and Read, 2008).

While water travels along the water potential gradient in roots by apoplastic and symplastic pathways (Steudle and Peterson, 1998), the presence of EcM fungi can drastically change the dynamics of plant water relations and root hydraulic properties, by altering both pathways, particularly around the root epidermal and cortical cells (Figure 1A; Marjanović et al., 2008; Smith and Read, 2008; Lehto and Zwiazek, 2011). Firstly, the presence of EcM fungi can substantially alter root anatomy and the hydrophilic properties of root apoplastic pathway (Nylund, 1987; Muhsin and Zwiazek, 2002). Secondly, the extensive hyphal network of mycorrhizas can significantly increase water supply toward the roots (Lehto and Zwiazek, 2011). Water availability in root cortex is subsequently altered. Root aquaporins – the water transporting Major intrinsic proteins (MIPs) located on cellular membranes – respond to the change in water availability, and rule the water permeability of symplastic pathway in root cortex and in endodermis where the apoplastic pathway is thought to be hindered by Casparian band (Figure 1A).

## Roles of Fungal Aquaporins in Ectomycorrhizal Root Water Transport

To understand root hydraulic properties of EcM plants, water transfer between both partners should be elucidated as a plant–fungi continuity. Theoretically, water can move in the extracellular space and in the cell walls of hydrophilic hyphae in a manner equivalent to the apoplastic pathway in plant roots. While in the hyphal protoplasts, water travels efficiently cross septa between adjacent cells with minimal barrier (Agerer, 2001; Peterson et al., 2004).

**FIGURE 2:**
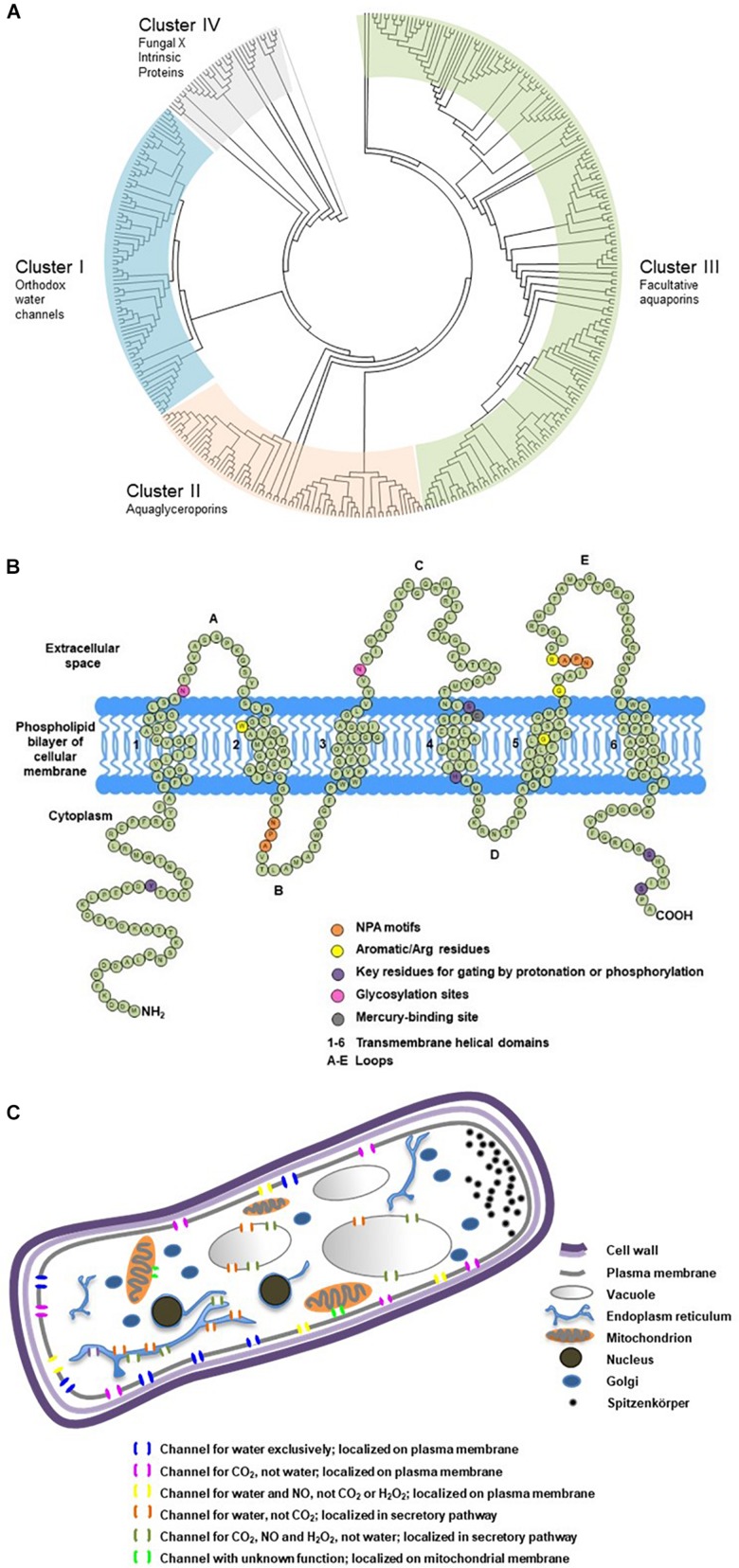
Phylogenetic classification, secondary structure and cellular localization of ectomycorrhizal fungal aquaporins. **(A)** Phylogenetic analysis of 376 fungal major intrinsic proteins (MIPs), including 152 sequences from 32 EcM fungi categorized into four clusters. The sequences were retrieved from public databases in JGI and NCBI. The classification referred to Xu et al. (2013). Divergence times for all branching points in the topology were calculated using the Maximum Likelihood method based on the JTT matrix-based model (Jones et al., 1992; Tamura et al., 2012) in MEGA 7 (Kumar et al., 2016). The tree was outgrouped to the aquaporin sequences of AqpZ from *Escherichia coli* and AQP1 from *Mus musculus*. **(B)** Transmembrane domains and signature motifs of fungal aquaporins. Protein secondary structure was predicted using SOSUI (Hirokawa et al., 1998). The diagram was reproduced from Xu (2015) with the author’s permission. **(C)** Possible subcellular localizations and transport functions of fungal aquaporins. Subcellular localization was predicted using TargetP (Emanuelsson et al., 2007). The diagram was modified from Xu (2015) with the author’s permission.

When water travels cross the cellular membrane of hyphal cells from apoplastic into symplastic space, or vice versa, its movement is regulated by an array of fungal MIPs with water permeability (Pettersson et al., 2005; Maurel and Plassard, 2011). These water channel proteins belong to the clusters of orthodox aquaporins or facultative aquaporins (Xu et al., 2013; Figure 2A), and are commonly referred as fungal aquaporins for their roles in determining water permeability of cellular membranes and water transport efficiency of symplastic pathway. They have 250–330 amino acids, forming six transmembrane domains, with three loops in the extracellular space, and two loops and two termini in the cytoplasm (Figure 2B). They also possess signature motifs for water selectivity and key residues for gating. Their subcellular localization on plasma membrane, secretory pathway or mitochondrial membrane can be predicted based on the motifs in the N-terminus (Figure 2C). The transport capacity can be predicted by how their sequences cluster into the phylogenetic relations, and be confirmed by functional assays of protein expression. A dozen of aquaporins have been functionally characterized in EcM fungus *Laccaria bicolor* and ectendomycorrhizal fungus *Terfezia claveryi*. The studies demonstrate their strong to moderate capacity of transporting water, urea, glycerol, ammonia, and CO_2_. Their expression can be altered by mycorrhization or abiotic cues such as drought, salt, low temperature, or pH (Dietz et al., 2011; Navarro-Ródenas et al., 2012, 2013; Xu et al., 2015), suggesting their multiple roles in plant–fungal interactions, and their involvement in water transport and nutrient transfer of the mycorrhizal partners. Differential expressions of fungal aquaporins were also observed in a cell-specific pattern, in EcM root tips, free-living mycelium, fungal mantle, Hartig net, and fruiting body. The accumulation of transcripts of *Tuber melanosporum* aquaporins in the mantle of mycorrhizal tips (Hacquard et al., 2013), the upregulation of several aquaporins in the formation of the fruiting body of *L. bicolor* (Xu et al., 2016), and the striking upregulation of two aquaporin genes of EcM *Cenococcum geophilum* induced by the symbiosis interaction with *Pinus silvestris* (Peter et al., 2016), are some examples of the physiological significance of fungal aquaporins in hyphal cells and at hypha-root interface.

Fungal aquaporins regulate the acquisition and release of water by the hyphae, and therefore play essential physiological roles in hyphal cell expansion, division, and hyphal fusion. Because of their significance in the processes of water entry and exit of the symplastic pathway, understanding their roles is key to elucidate the precise pathways for water transport from the fungal partner to the host roots in mycorrhizal associations. Firstly, fungal aquaporins regulate the amount of water acquired by the hyphae at mycelial front and subsequently transported into rhizomorphs. Secondly, the abundance and activity of fungal aquaporins could impact water availability in root extracellular space in Hartig net (Figures 1A,B). Ultimately, by changing the hydration in the apoplastic space, it can influence root aquaporin regulation and the overall root water uptake (Javot and Maurel, 2002; Marjanović et al., 2005; Lee et al., 2010; Dietz et al., 2011; Navarro-Ródenas et al., 2013; Xu et al., 2015; Calvo-Polanco et al., 2019).

The involvement of fungal aquaporins in root water transport was proven by the enhancement of root hydraulic conductance in *Picea glauca* roots mycorrhizal with the *L. bicolor* strains that overexpressed the fungal water-transporting aquaporins (Xu et al., 2015). As the conceptual model based on this study shows (Figure 1A), the contribution of *L. bicolor* hyphae to root water transport in *P. glauca* could involve increased apoplastic water transport in the root intercellular spaces, which may be attributed to water released from the hyphae and may lead to increased hydration at the fungal-root interface, and consequently impact aquaporin expression in mycorrhizal roots (Figure 1B2). Moderate increase in fungal aquaporin expression may lead to a further increase in hydration in the root intercellular space; therefore, apoplastic water transport in roots may be further enhanced (Figure 1B3). Under adverse conditions such as low temperatures, upregulation of fungal aquaporins to a furthest extent may lead to the increase in water influx into hyphal cells and the decrease in hydration in the root intercellular space, which consequently affects root aquaporin regulation and reduces root hydraulic conductance in the host plant. This suggests that the role of fungal aquaporins in EcM root water transport is highly dynamic and reversible. Further studies on the synchronization of fungal and plant aquaporins need to be conducted in the context of abiotic stresses, as well as under stimulation of stress-related and mycorrhiza-related phytohormones (Zargar et al., 2017; Lanfranco et al., 2018; Calvo-Polanco et al., 2019).

## Integrated Toolkits and Future Perspectives for Fungal Aquaporin Research

In the last two decades, aquaporin research has been accelerated substantially by the integrated approaches of bioinformatics, functional assays in singular cell expression systems, and molecular tools for abundance analysis and *in situ* visualization of aquaporin RNAs and proteins.

Phylogenetic reconstruction and peptide secondary structure prediction (Hirokawa et al., 1998; Emanuelsson et al., 2007) are usually the first steps and relatively rapid tools to screen putative fungal aquaporins that are retrieved from the expanding resource of sequenced fungal genomes. To date, Mycorrhizal Genomics Initiative (Martin et al., 2011, 2016; Nordberg et al., 2014; van der Heijden et al., 2015) has released the annotated genomes of more than 90 EcM fungal species (Figure 2A), which provides an unprecedented platform for *in silico* analysis of fungal MIPs and for selection of promising aquaporins of functional and physiological importance.

Functional assay can be considered as phenotyping of transmembrane porters. Using the singular cell expression systems of *Xenopus* oocytes and yeasts, researchers have unveiled diverse transport capacities of fungal aquaporins and postulated their versatile physiological functions (Figure 2C). One aquaporin may possess multiple transport functions, whereas different aquaporins may be permeable to similar substrates. Transport capacities of water and other small neutral molecules by fungal aquaporins, and their interaction with other transmembrane porters of amino acids, sugars and ions, are important areas to explore. Studies showed that a variety of fungal transmembrane porters were simultaneously upregulated by EcM interaction, which indicated the increased substrate exchange between the symbionts (Martin et al., 2010; Peter et al., 2016). In addition, the orchestrated gating and trafficking of aquaporins and other transmembrane porters may play important roles in cellular signaling; however, this area remains largely unexplored. The findings will contribute to the understanding of their multiple crucial roles in fungal growth and interaction with mycorrhizal plants (Nehls and Dietz, 2014; Verma et al., 2014).

Physiological roles of aquaporins with known transport capacities can be inferred by the alteration and synchronization of their expressions in response to abiotic stresses and stress-related phytohormones such as abscisic acid, cytokinins, salicylic acid, and strigolactones (Zargar et al., 2017; Lanfranco et al., 2018; Calvo-Polanco et al., 2019). The functional importance of aquaporins is also implied by the differential expressions of fungal aquaporins in extraradical mycelium, mantle and Hartig net, and of plant aquaporins in epidermis, cortex and endodermis (Figure 1A). In addition to RNA-seq differential expression analysis for the identification of significantly upregulated aquaporins (Martin et al., 2010; Peter et al., 2016), techniques such as qRT-PCR in combination with laser capture microdissection RNA extraction and mRNA *in situ* hybridization, allow researchers to examine the transcript abundance of plant aquaporins in different cell types along root water transport pathways (Almeida-Rodriguez and Hacke, 2012; Gambetta et al., 2013), and of fungal aquaporins in different hyphal compartments in mycorrhizal roots (Hacquard et al., 2013). In addition, transgenic fungal constructs that overexpress or silence water-transporting aquaporins, are powerful tools to confirm the physiological contributions of fungal aquaporins in mycorrhizal roots (Xu et al., 2015).

On a finer scale, advances in antibody and immunofluorescence techniques will facilitate the research on aquaporin subcellular localization and post-translational modifications such as phosphorylation, protonation and heteromerization. This will help to elucidate how the gating and trafficking mechanisms of fungal and plant aquaporins are linked to the changes in root hydraulic conductivity, as responses to mycorrhizal symbiosis, and abiotic cues.

## Data Availability Statement

The datasets generated for this study can be found in the JGI, https://mycocosm.jgi.doe.gov/Mycorrhizal_fungi/Mycorrhizal_fungi.info.html, by inquiring EcM fungal genomes with the keyword “aquaporin”.

## Author Contributions

HX and JZ conceptualized and wrote the manuscript.

## Conflict of Interest

The authors declare that the research was conducted in the absence of any commercial or financial relationships that could be construed as a potential conflict of interest.
